# Determination of the concentration of impurities in GaN from photoluminescence and secondary-ion mass spectrometry

**DOI:** 10.1038/s41598-020-59033-z

**Published:** 2020-02-10

**Authors:** M. A. Reshchikov, M. Vorobiov, O. Andrieiev, K. Ding, N. Izyumskaya, V. Avrutin, A. Usikov, H. Helava, Yu. Makarov

**Affiliations:** 1 Department of Physics, Virginia Commonwealth University, Richmond, VA 23220 USA; 2 Department of Electrical Engineering and Computer Science, Virginia Commonwealth University, Richmond, VA 23220 USA; 3 Saint-Petersburg National Research University of Information Technologies, Mechanics and Optics, 49 Kronverkskiy Ave., 197101 Saint Petersburg, Russia; 4 Nitride Crystals, Inc. 9702 Gayton Road, Ste. 320, Richmond, VA 23238 USA

**Keywords:** Semiconductors, Surfaces, interfaces and thin films

## Abstract

Photoluminescence (PL) was used to estimate the concentration of carbon in GaN grown by hydride vapor phase epitaxy (HVPE). The PL data were compared with profiles of the impurities obtained from secondary ion mass spectrometry (SIMS) measurements. Comparison of PL and SIMS data has revealed that apparently high concentrations of C and O at depths up to 1 µm in SIMS profiles do not represent depth distributions of these species in the GaN matrix but are rather caused by post-growth surface contamination and knocking-in impurity species from the surface. In particular, PL analysis supplemented by reactive ion etching up to the depth of 400 nm indicates that the concentration of carbon in nitrogen sites is below 2–5 × 10^15^ cm^−3^ at any depth of GaN samples grown by HVPE. We demonstrate that PL is a very sensitive and reliable tool to determine the concentrations of impurities in the GaN matrix.

## Introduction

GaN is a key material in modern solid-state lighting technology. The investigation and identification of point defects in GaN is very important, with immediate technology relevance to longer lifetime of light-emitting devices. Photoluminescence (PL) is a powerful tool for investigation of point defects in GaN^1^. Only few point defects in unintentionally doped GaN are well understood and reliably identified. One of them is the C_N_ defect with the −/0 and 0/ + thermodynamic transition levels at 0.916 eV and ~0.3 eV, respectively, above the valence band^2–5^. This defect is responsible for the yellow luminescence (YL1) band with a maximum at 2.2 eV and zero phonon line (ZPL) at 2.59 eV in *n*-type GaN. Transitions via the 0/ + level of this defect can be observed as the blue luminescence (BL_*C*_) band with a maximum at 3.3 eV at high excitation intensity^4^. The YL1 band is commonly observed in *n*-type GaN layers grown by metalorganic chemical vapor deposition (MOCVD), where the concentration of unintentionally introduced carbon is usually in the range of 10^16^–10^18^ cm^−3^, depending on growth conditions^6,7^. Undoped or Si-doped GaN samples grown by hydride vapor phase epitaxy (HVPE) contain much less carbon (10^15^–10^16^ cm^−3^)^8–11^, yet still the YL1 band may be strong in these samples due to high hole-capture cross-section for the C_N_ defects^12^.

We have recently demonstrated^12^ that PL can be used for determination of concentrations of defects responsible for PL bands. However, the concentrations of impurities found from PL were inconsistent with the concentrations found from secondary-ion mass spectrometry (SIMS) analysis in that study. Comparison of these two techniques is complicated by the following ambiguity. SIMS measurements provide depth profiles of impurity concentrations regardless of positions of impurity species (lattice sites or precipitations), whereas PL signal, excited with above-bandgap light, is collected from impurity atoms at lattice sites in the near-surface region (roughly, top 0.4 µm layer in GaN). The problem is that close to the surface, where PL is collected, very high concentrations of carbon and oxygen are sometimes observed in SIMS profiles^8,10^, and it is not clear if these high concentrations really represent the substitutional point defects in crystal lattice or impurities accumulated at structural defects. Moreover, high concentrations of certain impurities in the near-surface region can be a result of knocking-in the surface contaminants into material volume during sputtering with a primary ion beam. In MOCVD-grown GaN samples, these “tails” of impurity concentrations from the surface to bulk are typically short (less than 50 nm)^7,13,14^. However, in HVPE-grown GaN, the tails of Si, O, C, and H are typically longer (200–500 nm)^8,10^.

The near-surface tailing in SIMS profiles, in particular for C, O, and H, can be explained by kick-on of the impurity atoms from the surface contaminations into the depth by the primary ion beam; however, this is expected to cause a surface peak of no more than 20–50 nm. A possible explanation for impurity tails extending deeper is the segregation of impurities at the surface during growth and partial out diffusion through grain boundaries, dislocations (particularly open-core screw dislocations in GaN) after the growth termination^11^. Moreover, high surface roughness can lead to apparent long impurity tails in SIMS depths profiles that do not represent an actual chemical distribution but are merely an artifact^15^. Sometimes, significant accumulation of impurities is observed, which may create a highly-conductive layer near the surface^16^.

In this work, we have analyzed depth distributions of carbon and other impurities (H, Si, O) in HVPE-grown GaN layers by using SIMS and PL techniques. Depth distribution of carbon in nitrogen lattice sites (C_N_) was evaluated from PL measurement of as-grown films and after removal of up to 400 nm by inductively-coupled plasma reactive ion etching (ICP-RIE). Comparison of the PL data with SIMS depth profiles indicates that the concentration of the C_N_ defect, which is responsible for YL1 band, is low and uniform for the studied GaN samples. This finding may indicate that SIMS profiles in the near-surface region predominantly represent post-growth impurity accumulation at the surface and/or structural defects, such as grain boundaries and threading dislocations.

## Results and Discussion

### Secondary-ion mass spectrometry

**Figure 1:**
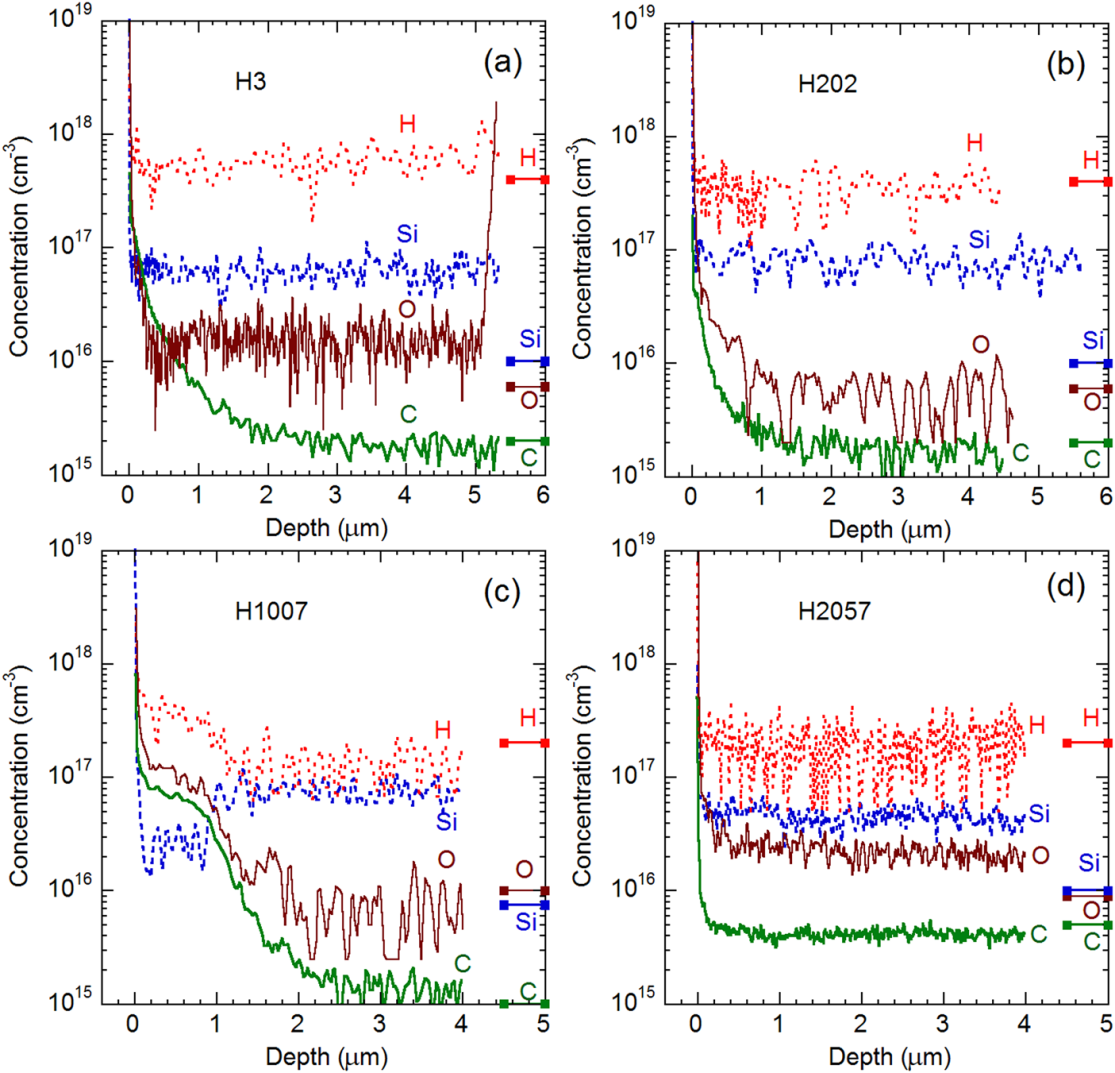
Impurity profiles for H, Si, O, and C from SIMS measurements in GaN samples H3 (**a**), H202 (**b**), H1007 (**c**), and H2057 (**d**). Bars on the right side indicate detection limits for these impurities.

Depth profiles for H, Si, O, and C impurities in the HVPE-grown GaN samples are shown in Fig. 1. The most interesting feature, to be analyzed in this work, is significant tailing of carbon concentration from the surface into depth for some samples. At depth, *d*, exceeding 1–2 µm, the concentration of C is below the detection limit of SIMS (2 × 10^15^ cm^−3^ for samples H3, H202, and H1007, and 5 × 10^15^ cm^−3^ for sample H2057), in agreement with our expectations for undoped GaN grown by HVPE. However, at *d* < 0.4 µm, the concentration of C is at least an order of magnitude higher in all the samples except for H2057. Oxygen shows similar tailing, while the concentration of Si reaches the saturation level within the first 200 nm, with no further tailing. The concentration of hydrogen is below the detection limit (2–5 × 10^17^ cm^−3^) in these samples. Note that the total concentration of O and Si (which are shallow donors in GaN) in the bulk region is close to the total concentration of shallow donors determined from temperature-dependent Hall effect (about 1 × 10^17^ cm^−3^ in all the samples)^17^. Table 1 summarizes the SIMS results for the C, O, and Si impurities. The data for the first 100 nm is ignored, because surface contamination may severely affect the measured concentration in the near-surface region. Two regions (*d* = 0.1–0.4 µm and *d* = 2–4 µm) are selected to show the difference between the near-surface region from where PL signal is collected and the bulk region where the role of the surface contamination is clearly negligible.

**Table 1:** Average concentrations of impurities in near-surface (*d* = 0.1–0.4 µm) and bulk (*d* = 2–4 µm) regions of GaN from SIMS measurements.

Sample	[C] (cm^−3^)		[O] (cm^−3^)		[Si] (cm^−3^)	
	*d* = 0.1–0.4 µm	*d* = 2–4 µm	*d* = 0.1–0.4 µm	*d* = 2–4 µm	*d* = 0.1–0.4 µm	*d* = 2–4 µm
H3	5.1 × 10^16^	<2 × 10^15^	3.1 × 10^16^	1.6 × 10^16^	6.8 × 10^16^	6.5 × 10^16^
H202	1.3 × 10^16^	<2 × 10^15^	3.1 × 10^16^	<6 × 10^15^	8.6 × 10^16^	8.0 × 10^16^
H1007	8.2 × 10^16^	1.5 × 10^15^	1.3 × 10^17^	<1 × 10^16^	2.3 × 10^16^	7.5 × 10^16^
H2057	<5 × 10^15^	<5 × 10^15^	3.5 × 10^16^	2.1 × 10^16^	4.8 × 10^16^	4.5 × 10^16^

The surface tailing in SIMS chemical profiles could represent impurity concentration in the matrix, or it may be caused by impurity segregation at grain boundaries and dislocations, as well as by secondary implantation of the surface contaminants. Detailed analysis of PL may help to resolve this ambiguity, since PL signal is collected from near-surface region only, up to ~0.4 µm in GaN. Indeed, the absorption coefficient *α* in GaN at 3.81 eV (the 325 nm line of HeCd laser) is 1.2 × 10^5^ cm^−1^^18^. Then, 91% of light is absorbed in the first 0.2 µm from the surface, and less than 1% reaches depths beyond 0.4 µm. The diffusion length of charge carriers is typically less than 0.3 µm in GaN^19,20^. In this near-surface region, the concentration of C appears to be very high in samples H3, H202, and H1007 (Fig. 1 and Table 1). Then, if the C impurity tail represents carbon atoms incorporated into GaN matrix, the concentration of the C_N_ defects obtained from PL experiments should also be very high.

### Photoluminescence from as-grown GaN

**Figure 2:**
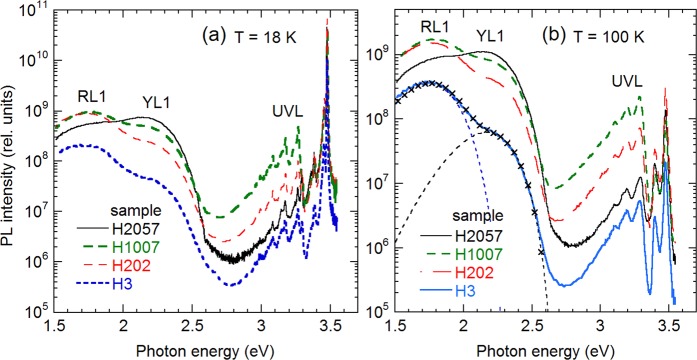
PL spectra from GaN samples measured at *T* = 18 K (**a**) and *T* = 100 K (**b**). *P*_*exc*_ = 10^−4^ W/cm^2^. All the measurements are done in identical conditions. The PL intensity is multiplied by *λ*^3^ to present PL intensity as the number of emitted photons versus photon energy (see Methods). The thin dashed lines in (**b**) show RL1 and YL1 band shapes simulated by using Eq. (1) with the following parameters: *I*_max_ = 3.5 × 10^8^, *S* = 10, *E*_0_^*^ = 2.35 eV, *ħω*_max_ = 1.75 eV for the RL1 band and *I*_max_ = 6.2 × 10^7^, *S* = 7, *E*_0_^*^ = 2.67 eV, *ħω*_max_ = 2.17 eV for the YL1 band. The “ × ” symbols show the sum of the simulated bands.

PL spectra measured at 18 and 100 K from as-grown GaN samples are shown in Fig. 2. At *T* = 18 K (Fig. 2a), excitonic emission contains lines usual for undoped GaN^1^, with the strongest peak attributed to donor-bound exciton (DBE). The DBE line, with a full width at half maximum (FWHM) of about 3 meV, has a maximum between 3.475 and 3.479 eV in different samples, slightly blue-shifted from its position in bulk GaN (about 3.471 eV) due to strain in GaN layers grown on sapphire substrates. The defect-related emission is represented by three bands in these samples: the ultraviolet luminescence (UVL) band with the strongest peak at 3.27 eV followed by several LO phonon replicas, the yellow luminescence (YL1) band with a maximum at 2.2 eV, and the red luminescence (RL1) band with a maximum at 1.8 eV. The characteristics of the YL1 band, namely the shape, position, and fine structure with the ZPL at 2.59 eV, are the same in all the samples. This allows us to attribute it with high confidence to the C_N_ defect^3,4^. The UVL band is attributed to the Mg_Ga_ defect^21^. The chemical identity of the RL1 band remains unknown, yet it is attributed to transitions from the conduction band (or from shallow donors) to a deep acceptor^17^. The transition level of this acceptor is located at 1.1–1.2 eV above the valence band.

With increasing temperature, the exciton emission is quenched due to dissociation of excitons (Fig. 2b), and transitions from shallow donors to the Mg_Ga_, C_N_, and the RL1-related acceptors are gradually replaced with transitions from the conduction band to the same acceptors. At *T* = 100 K, the PL decay of the UVL, YL1, and RL1 bands after a laser pulse becomes nearly exponential, which is consistent with transitions from the conduction band to acceptor levels in *n*-type GaN. The PL lifetime, *τ*, at *T* = 100 K for samples analyzed in Fig. 2 is 10–20 µs (UVL), 290–520 µs (YL1), and 720–1600 µs (RL1), which corresponds to the concentration of free electrons in the range of (1.6−3.2) × 10^16^ cm^−3^ according to the electron-capture coefficients found in ref. ^17^. In contrast, the PL decay at *T* = 18 K is nonexponential, which is consistent with donor-acceptor pair (DAP) transitions involving shallow donors. The intensity of the RL1 and YL1 bands increases by about a factor of 2 with increasing temperature from 18 to 100 K in the studied samples. The increase occurs simultaneously with the quenching of the exciton emission and can be explained by redistribution of recombination flows in *n*-type GaN^22^. The shapes of broad PL bands can be simulated with the following equation obtained by using one-dimensional configuration coordinate model^23^.

1 $${I}^{PL}(\hslash \omega )={I}_{\max }^{PL}\exp \,[-2{S}_{e}{(\sqrt{\frac{{E}_{0}^{\ast }-\hslash \omega }{{E}_{0}^{\ast }-\hslash {\omega }_{\max }}}-1)}^{2}],$$

where ${I}_{\max }^{PL}$ is the intensity of a PL band at its maximum, *S*_*e*_ is the Huang-Rhys factor for the excited state (when an acceptor binds a hole), $\hslash \omega$ and $\hslash {\omega }_{\max }$ are the photon energy and position of the band maximum, respectively; ${E}_{0}^{\ast }={E}_{0}+0.5\hslash \Omega$, *E*_0_ is the ZPL energy, and $\hslash \Omega$ is energy of the dominant phonon mode in the excited state.

**Figure 3:**
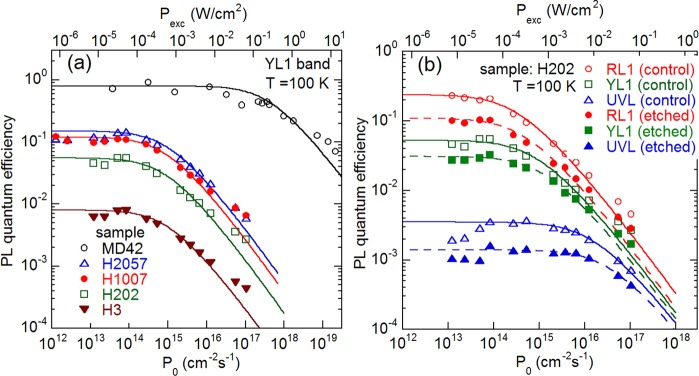
Dependence of quantum efficiency of PL on excitation intensity at *T* = 100 K. (**a**) The YL1 band in different samples. (**b**) RL1, YL1, and UVL bands in control (unetched) and 400 nm-etched areas of sample H202. The lines are calculated using Eq. (2) with parameters given in Table 2 and with τ = 25 µs (sample MD42), 290 µs (H2057), 520 µs (H1007), 300 µs (H202), and 450 µs (H3) for the YL1 band in (**a**) and with *τ* = 880 µs (RL1), 300 µs (YL1), and 10 µs (UVL) for sample H202 in (**b**).

The concentration of defects, *N*, responsible for the UVL, YL1 and RL1 bands can be determined at 100 K from the dependence of the absolute internal quantum efficiency (IQE) of PL, *η*, on the excitation intensity, *P*_*exc*_, or the excitation photon flux, *P*_0_, as explained elsewhere^12,24,25^. The $\eta ({P}_{0})$ dependence can be fitted with the following expression

2 $$\eta ({P}_{0})={\eta }_{0}\frac{{P}_{1}}{{P}_{0}}\,\mathrm{ln}(1+\frac{{P}_{0}}{{P}_{1}}),$$

where ${P}_{1}=N{(\alpha \tau {\eta }_{0})}^{-1}$, *α* is the absorption coefficient of GaN for incident photons (1.2 × 10^−5^ cm^−1^ at 325 nm), *τ* is PL lifetime, and *η*_0_ is the IQE in the limit of low excitation intensities. The quantum efficiency is constant when ${P}_{0}\ll {P}_{1}$, and it decreases when ${P}_{0} > {P}_{1}$. The $\eta ({P}_{0})$ dependences for the YL1 band in several GaN samples, including one MOCVD GaN sample with high concentration of C^4^, are shown in Fig. 3 where they are fitted with Eq. (2).

**Table 2:** Absolute IQE of defect-related PL at *T* = 100 K and the concentration of defects responsible for PL bands in GaN.

Sample	RL1 band		YL1 band (C_N_)		UVL band (Mg_Ga_)	
	*η*_0_	*N* (cm^−3^)	*η*_0_	*N* (cm^−3^)	*η*_0_	*N* (cm^−3^)
H3	0.055	1.5 × 10^15^	0.008	3.5 × 10^14^	0.00033	8 × 10^12^
H3 (400 nm – etched)	0.048	1.2 × 10^15^	0.008	4 × 10^14^	0.00032	8 × 10^12^
H202	0.24	4 × 10^15^	0.053	7 × 10^14^	0.0035	3 × 10^13^
H202 (400 nm – etched)	0.11	2.6 × 10^15^	0.031	8 × 10^14^	0.0014	2.8 × 10^13^
H1007	0.24	8 × 10^15^	0.11	3 × 10^15^	0.011	2 × 10^14^
H2057	0.09	3 × 10^15^	0.14	4 × 10^15^	0.006	6 × 10^12^
MD42			0.8	5 × 10^17^	0.0003	~10^14^

Note that the values for samples H3 and H202 in Table 2 differ from those reported for the same samples in ref. ^12^. The main reason is that in the current work PL spectra measured as a function of wavelength *λ* were multiplied by *λ*^3^ to represent PL spectra in units proportional to the number of emitted photons as a function of photon energy (see Methods). As a result of this omission in our publications prior to ref. ^4^, the concentration of defects responsible for the RL1 band was underestimated by a factor of 2 and that for the UVL band was overestimated by a factor of 3 in ref. ^12^. In addition, after 2–4 years following the initial analysis of these samples, the PL intensity and spectrum for sample H3 have changed, which explains the remaining difference by up to a factor of 3 between the concentrations for sample H3 reported in ref. ^12^ and in the current work.

Equation (2) contains only one fitting parameter, *N*, because *τ* for a particular PL band is found directly from time-resolved PL measurements, while *η* (and *η*_0_) is determined from comparison of integrated over PL band intensity with that from calibrated GaN samples (see Methods). We estimate that the absolute value of *η*_0_ (and, therefore *N*) may have an error of up to half order of magnitude, mostly because of different and unknown light extraction efficiencies in different samples, including the calibrated samples^12^. However, the relative values of *η*_0_ (and *N*) for a given sample are determined with better accuracy.

Concentrations of defects responsible for the RL1, YL1, and UVL bands in GaN samples, as found from the fit of the experimental data at *T* = 100 K with Eq. (2), are given in Table 2.

We can see from Table 2 that the concentration of the C_N_ in HVPE GaN determined from PL data is significantly lower than the concentration of carbon impurities in the 0.4 µm-thick near-surface layer derived from the SIMS analysis (Fig. 1 and Table 1). In contrast, for GaN grown by MOCVD (sample MD42 in Table 2), the concentration of the C_N_ defects calculated from PL data is nearly identical to the concentration of carbon obtained from SIMS analysis in earlier work^4^. To further clear up the question whether the SIMS tailing in HVPE GaN represents carbon incorporated into GaN lattice, we conducted etching of GaN layers to different depths and repeated PL measurements.

### Effect of etching on photoluminescence

**Figure 4:**
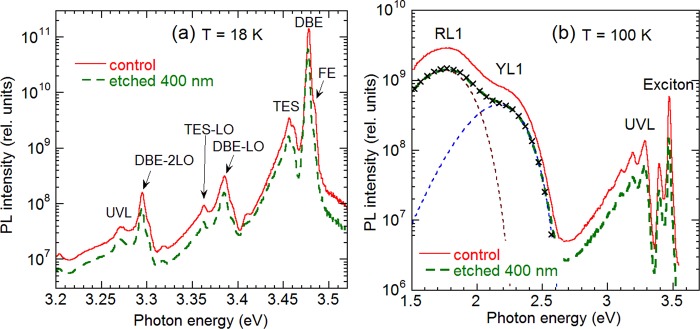
PL spectra from sample H202 before and after etching that removed 400 nm. (**a**) *T* = 18 K, *P*_*exc*_ = 0.1 W/cm^2^; (**b**) *T* = 100 K, *P*_*exc*_ = 10^−4^ W/cm^2^. Free exciton (FE), donor-bound exciton (DBE), two-electron satellites (TES) lines and their LO phonon replicas are identified at *T* = 18 K. The thin dashed lines in (**b**) show RL1 and YL1 band shapes simulated by using Eq. (1) with *I*_max_ = 2.77 × 10^9^ (RL1) and *I*_max_ = 7.85 × 10^8^ (YL1). The rest parameters are the same as in the caption to Fig. 2. The × symbols show the sum of the simulated bands.

Some areas of selected samples were etched to depths of 100 and 400 nm by ICP-RIE. In these experiments, samples H3 and H202 were studied in greater detail, because the carbon concentration in their SIMS profiles drops dramatically between 0.2 and 1 μm (Fig. 1). PL spectra from the unetched area and a 400 nm-etched area in sample H202 are compared in Fig. 4. The PL spectra before and after the etching are identical, and only the PL intensity decreased by a factor of 2. Even smaller difference is observed between unetched and 100 nm-etched areas of this sample. In sample H3, the YL1 band intensity did not change after removal of 100 and 400 nm by etching, whereas the UVL intensity increased by a factor of 3.5 at 100 nm and returned to the initial value at 400 nm. Note that PL intensity, or PL IQE, is not necessarily proportional to the concentration of related defects, because nonradiative recombination efficiency may change with depth or as a result of plasma etching. The fact that intensities of all PL bands change by the same factor for sample H202 indicates that the change is caused by increased contribution of nonradiative recombination rather than by decreased concentrations of radiative defects.

The concentrations of radiative defects after the etching were also estimated from the dependence of the IQE of PL on the excitation intensity. Figure 3(b) shows the $\eta ({P}_{0})$ dependences for PL bands in sample H202 before and after etching. The concentrations of defects responsible for the RL1, YL1, and UVL bands in unetched and 400-nm etched areas of samples H3 and H202, found from the fit of experimental data with Eq. (2), are given in Table 2. The PL analysis at different depths shows no significant changes in concentrations of C_N_, Mg_Ga_ and RL1-related acceptor in the HVPE-grown GaN samples.

From comparison of PL data shown in Tables 1 and 2 and in Figs. 3 and 4, we conclude that the concentration of the C_N_ defects is equal to or below the detection limit of SIMS for undoped GaN layers grown by HVPE. More importantly, the concentration of the C_N_ determined from the PL measurements does not change with depth, and it is the same low in the near-surface region and in the bulk. This means that the impurity tailing from the surface observed in SIMS profiles of some samples (Fig. 1) do not represent carbon in nitrogen sites of the GaN lattice. Note that the profiles of C, O, and H are similar (best seen in Fig. 1(c)), which may further confirm that organic contaminants at the surface are responsible for the long impurity tails in the SIMS profiles.

In summary, comparison of PL and SIMS data has revealed that apparently high carbon concentration at depths up to 1 µm in SIMS profiles does not represent the depth distribution of carbon in lattice sites of the GaN matrix. The PL data obtained from as-grown samples and after removal of up to 400 nm by ICP-RIE etching indicate that the concentration of carbon in nitrogen sites of HVPE-grown GaN is below the detection limit of SIMS in bulk and near-surface regions. The near-surface impurity tails in SIMS depth profiles are likely caused by post-growth contamination of sample surfaces followed by their knocking-in or/and impurity accumulation at structural defects (including rough surface, grain boundaries, and dislocations). The PL technique is demonstrated as a sensitive and relatively accurate tool to determine the concentrations of radiative defects such as C and Mg in GaN.

## Methods

### Samples

Unintentionally doped GaN layers, 7–24 μm-thick, were grown by HVPE on *c*-plane sapphire substrates. The concentration of free electrons in these samples is about 1 × 10^17^ cm^−3^ at room temperature, as determined from the temperature-dependent Hall effect and time-resolved PL measurements^17^. One GaN sample (MD42) from Institut für Physik, Magdeburg, Germany^4^, was grown on sapphire by MOCVD and doped with Si and C. The partial etching of GaN was carried out in a Samco inductively coupled plasma (ICP) etching system (model: RIE-101 iPH) with photoresist spr955 serving as a mask. The etching rate was about 40 nm/min under a gas mixture condition of Cl_2_/SiCl_4_/Ar = 30/5/18 sccm, with the ICP source power and bias power set to 80/30 W and chamber pressure of 0.6 Pa. The etching depth was confirmed by the Veeco Dektak 150 surface profiler.

### Experimental details

Steady-state PL was excited with an unfocused He-Cd laser (30 mW, 325 nm), dispersed by a 1200 rules/mm grating in a 0.3 m monochromator and detected by a cooled photomultiplier tube. Calibrated neutral-density filters were used to attenuate the excitation intensity (*P*_exc_) over the range 10^–7^ – 0.2 W/cm^2^. Time-resolved PL was excited with a pulsed nitrogen laser (pulses with duration of 1 ns and repetition frequency of 6 Hz, and photon energy of 3.68 eV) and analyzed with an oscilloscope. A closed-cycle optical cryostat was used for temperatures between 15 and 320 K. All the samples were studied under identical conditions. The measured PL spectra were corrected for the optical elements and detector sensitivities with a calibrated tungsten-halogen lamp and were also multiplied by *λ*^3^ in order to plot the spectra in units proportional to the number of emitted photons as a function of photon energy^4^. The absolute internal quantum efficiency of PL, *η*, is defined as $\eta ={I}^{PL}/G$, where *I*^*PL*^ is the integrated PL intensity from a particular PL band and *G* is the concentration of electron-hole pairs created by the laser per second in the same volume. To find *η* for a particular PL band, we compared its integrated intensity with the PL intensity obtained from a calibrated GaN sample^22,26^.

SIMS measurements of HVPE-grown GaN sample have been carried out by the Evans Analytical Group. A very low detection limit for carbon was achieved in vacuum by removing carbon adsorbed at the surface. The reduction in surface carbon resulted in less interference (and thus a lower background/detection limit) during the SIMS measurement of the underlying GaN region. Note that the detection limit is not the same for different samples because the studied samples were sent for SIMS analysis at different times.
